# An unprecedented dioxygen species revealed by serial femtosecond rotation crystallography in copper nitrite reductase

**DOI:** 10.1107/S2052252517016128

**Published:** 2018-01-01

**Authors:** Thomas P. Halsted, Keitaro Yamashita, Kunio Hirata, Hideo Ago, Go Ueno, Takehiko Tosha, Robert R. Eady, Svetlana V. Antonyuk, Masaki Yamamoto, S. Samar Hasnain

**Affiliations:** a Molecular Biophysics Group, Institute of Integrative Biology, Faculty of Health and Life Sciences, University of Liverpool, Liverpool L69 7ZB, England; b SR Life Science Instrumentation Unit, RIKEN SPring-8 Centre, Sayo 679-5148, Japan; c Biometal Science Laboratory, RIKEN SPring-8 Centre, Sayo 679-5148, Japan

**Keywords:** serial femtosecond rotation crystallography, O_2_ binding, copper nitrite reductase

## Abstract

Synchrotron-based X-ray structural studies of ligand-bound enzymes are powerful tools to further our understanding of reaction mechanisms. For redox enzymes, it is necessary to study both the oxidized and reduced active sites to fully elucidate the reaction, an objective that is complicated by potential X-ray photoreduction. In the presence of the substrate, this can be exploited to construct a structural movie of the events associated with catalysis. Using the newly developed approach of serial femtosecond rotation crystallography (SF-ROX), an X-ray damage-free structure of the as-isolated copper nitrite reductase (CuNiR) was visualized. The sub-10 fs X-ray pulse length from the SACLA X-ray free-electron laser allowed diffraction data to be collected to 1.6 Å resolution in a ‘time-frozen’ state. The extremely short duration of the X-ray pulses ensures the capture of data prior to the onset of radiation-induced changes, including radiolysis. Unexpectedly, an O_2_ ligand was identified bound to the T2Cu in a brand-new binding mode for a diatomic ligand in CuNiRs. The observation of O_2_ in a time-frozen structure of the as-isolated oxidized enzyme provides long-awaited clear-cut evidence for the mode of O_2_ binding in CuNiRs. This provides an insight into how CuNiR from *Alcaligenes xylosoxidans* can function as an oxidase, reducing O_2_ to H_2_O_2_, or as a superoxide dismutase (SOD) since it was shown to have ∼56% of the dismutase activity of the bovine SOD enzyme some two decades ago.

## Abstract 2

The observation of O_2_ in a time-frozen structure using serial femtosecond rotation crystallography of the as-isolated oxidized enzyme provides long-awaited clear-cut evidence for the mode of O_2_ binding in CuNiRs. This provides an insight into how CuNiR from *A. xylosoxidans* can function as an oxidase, reducing O_2_ to H_2_O_2_, or as a superoxide dismutase (SOD) since it was shown to have ∼56% of the dismutase activity of the bovine SOD enzyme some two decades ago.

## 1. Introduction   

Throughout the 20th century, the increasing use of nitrogen fertilizers to boost food production has resulted in an imbalance of the nitrogen cycle, leading to the accumulation of higher nitrogen oxide levels in soil and surface waters (Gruber & Galloway, 2008 ▸; Canfield *et al.*, 2010 ▸). Denitrifying microbes utilize these nitrogen oxides as electron acceptors in the anaerobic/aerobic oxidation of organic matter, or less frequently as an inorganic source of electrons. This respiratory pathway usually has N_2_ as the final product, but the greenhouse gas N_2_O is a significant byproduct in this process, impacting both agricultural productivity and the environment. The reduction of nitrite to form NO by nitrite reductase (NiR) is a key step in this process because it is at this point that the loss of terrestrial fixed nitrogen to the atmosphere occurs. This reaction is carried out by two distinct types of highly conserved dissimilatory NiRs (Zumft, 1997 ▸; Tavares *et al.*, 2006 ▸). The homodimeric *cd*_1_-haem NiRs are one type, while the second type consists of the homotrimeric copper nitrite reductases (CuNiRs) (Fülöp *et al.*, 1995 ▸; Kakutani *et al.*, 1981 ▸). Extensive structural studies have shown that each CuNiR monomer contains two copper sites: one type 1 copper (T1Cu) site and one type 2 copper (T2Cu) catalytic site (Godden *et al.*, 1991 ▸). The T1Cu site accepts an electron from a carrier protein, either azurin or a *c*-type cytochrome, and in some CuNiRs these are present in an additional tethered domain (Antonyuk *et al.*, 2013 ▸).

Numerous crystallographic studies of CuNiRs have shown the catalytic type 2 Cu sites to be located at the interface between adjacent monomers and to typically have a Cu–(His)_3_–H_2_O ligation (Godden *et al.*, 1991 ▸). In some structures an additional H_2_O or an anion ligand from the crystallization solution is observed (Fukuda *et al.*, 2016 ▸). The active-site pocket has an Asp_CAT_ and a His_CAT_ residue, which are conserved in all CuNiRs and which mutational studies have shown to be essential for effective catalysis (Kataoka *et al.*, 2000 ▸). These residues are linked to the catalytic centre *via* a water bridge. During enzyme turnover nitrite binds to the T2Cu ion *via* O atoms in an asymmetric bidentate fashion, replacing a bound water molecule (Tocheva *et al.*, 2004 ▸). Proteins with catalytic redox metals are prone to reduction during synchrotron-radiation crystallography (SRX; Garman & Weik, 2015 ▸). In the case of CuNiRs this reduction generates electrons that drive enzyme turnover *in crystallo* (Hough *et al.*, 2008 ▸) and has been exploited by performing low-dose serial crystallography on a single crystal to observe intermediate states in the conversion of nitrite to NO (Horrell *et al.*, 2016 ▸). These atomic resolution structures revealed two conformations of the bound nitrite at a low initial accumulated radiation dose of 0.69 MGy that subsequently stabilized to a single ‘side-on’ conformation after 2.76 MGy. This indicates the high sensitivity of CuNiR to exposure to X-ray-induced changes at the T2Cu site. Such radiation-induced chemistry can be avoided using X-ray free-electron lasers, which produce very high intensity, extremely short duration (femtosecond) pulses of X-rays. This recently developed technique opened up the unique possibility of recording diffraction data on a timescale that is shorter than the molecular vibrational (10^−10^ s) and rotational (10^−13^ s) motions and provides time-frozen structures prior to the onset of any molecular motion or reaction induced by X-rays (Dantus *et al.*, 1990 ▸).

As well as reducing nitrite, some CuNiRs have an oxidase activity (∼3% of that of nitrite reduction) that results in the reduction of O_2_ to form H_2_O_2_, leading to inactivation of the enzyme unless catalase is present (Zumft, 1997 ▸; Kakutani *et al.*, 1981 ▸). The NiR function of typical CuNiRs has been extensively studied, but studies of their interaction with dioxygen reduction are scarce. It was recognized some two decades ago that the T2Cu centre of CuNiR shares structural similarity with the active site of Cu,Zn-superoxide dismutase (SOD) and that *Alcaligenes xylosoxidans* CuNiR (*Ax*NiR) had 56% of the catalytic activity of bovine SOD, which catalyzes one of the most rapid enzymatic reactions in biology (*k*_cat_/*K*_m_ ≃ 7 × 10^9^ *M* ^−1^ s^−1^; Strange *et al.*, 1995 ▸). *Ax*NiR also exhibits an oxidase activity and it is reasonable to expect ligation of 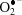 at the T2Cu. However, despite the determination of numerous structures of *Ax*NiR using synchrotron radiation from cryogenically maintained crystals, O_2_ bound to the T2Cu has never been observed. In a recent X-ray spectroscopic study of the oxidase-positive NiR from *Geobacillus thermodenitrificans* (*Gt*NiR), radiation-induced chemistry was associated with the observation of a diatomic ligand, suggested to be side-on-bound dioxygen (Fukuda *et al.*, 2015 ▸). Detection of this species has been suggested to arise from high thermal stability of the enzyme coupled with X-ray-induced reduction of T1Cu, resulting in electron transfer (ET) to T2Cu.

NiR from *A. faecalis* (*Af*NiR) has recently been the subject of an extensive investigation by serial femtosecond crystallo­graphy (SFX; Spence *et al.*, 2012 ▸; Schlichting, 2015 ▸), which used over 80 000 microcrystals passed through an injector to capture diffraction images upon interaction with the X-ray free-electron laser (XFEL) femtosecond pulses (Fukuda *et al.*, 2016 ▸). For *Af*NiR in the resting-state structure, a chloride ion originating from the purification protocol used for this enzyme was found to be ligated to T2Cu. While SFX can be utilized to visualize time-resolved systems (Young *et al.*, 2016 ▸; Barends *et al.*, 2015 ▸) and proteins that are difficult to crystallize (Kang *et al.*, 2015 ▸), the small crystal volume scales negatively with high-resolution data collection. Here, we have utilized serial femtosecond rotation crystallography (SF-ROX) using 64 large blue crystals to visualize the resting state of the active site of *Ax*NiR, one of the most well studied CuNiRs (Hirata *et al.*, 2014 ▸). To provide a direct comparison with the SRX structure, data were obtained from a crystal grown in the same batch that was cryogenically maintained on BL41XU at SPring-8. The SRX structure revealed the expected water ligand bound to the catalytic T2Cu centre, while the SF-ROX structure showed an unprecedented dioxygen ligand bound to the T2Cu site in the resting state of the aerobically purified as-isolated enzyme.

## 2. Methods   

### 2.1. Cloning and overexpression   

A DNA fragment encoding the ORF for the wild-type NiR gene from *A. xylosoxidans* (*nirK*) without its periplasmic signal peptide was cloned into a pET-28a(+) vector (Novagen, San Diego, California, USA). The gene was amplified by PCR using the oligonucleotide primers *Ax*NiR_fw (5′-CCCGTCTCCCATGCAGGACGCCGACAAGC-3′) and *Ax*NiR_rv (5′-GGAAGCTTTCAGCGCGGAATCGGC-3′) (restriction sites are underlined), and cloned between the HindIII and NcoI restriction sites to give the recombinant vector pET-28a(+)-*Ax*NiR-wt for overexpression. pET-28a(+)-*Ax*NiR-wt was transformed into competent *Escherichia coli* BL21 (DE3) cells using heat shock. The transformant was cultured on Kan^R^ Lysogeny Broth (LB) agar to isolate individual colonies. 1 l LB supplemented with 50 µg ml^−1^ kanamycin was inoculated with a single colony and incubated with shaking at 37°C. Protein overexpression was induced with 0.5 m*M* isopropyl β-d-1-thiogalactopyranoside (IPTG) and 1 m*M* CuSO_4_.

### 2.2. Purification   

Incubation continued for 24 h before the cells were harvested by centrifugation. The cell pellets were resuspended in 20 m*M* Tris–HCl pH 7.0, 0.1 mg ml^−1^ lysozyme and were disrupted by sonication. The cell lysate was dialyzed against 20 m*M* Tris–HCl pH 7.0, 0.1 m*M* CuSO_4_ to restore the copper content of the T2Cu site (Libby & Averill, 1992 ▸). The lysate was applied onto a carboxymethyl cellulose column (Whatman, Kent, England) pre-equilibrated with five column volumes of 20 m*M* Tris–HCl pH 7.0. The column was then washed with an increasing NaCl gradient in 20 m*M* Tris–HCl pH 7.0. *Ax*NiR was eluted in the 100 m*M* NaCl fraction, concentrated and loaded onto a GE HiLoad 16/600 Superdex 200 size-exclusion chromatography (SEC) column pre-equilibrated with 200 m*M* NaCl in 20 m*M* Tris–HCl buffer pH 7.0. *Ax*NiR eluted at 74.5 ml. The protein purity was confirmed by SDS–PAGE and *Ax*NiR was stored at −80°C in SEC buffer. The protein concentration was determined using an extinction coefficient of ∊_280_ = 43.89 m*M* ^−1^ cm^−1^.

### 2.3. Crystallization   

**Figure 1:**
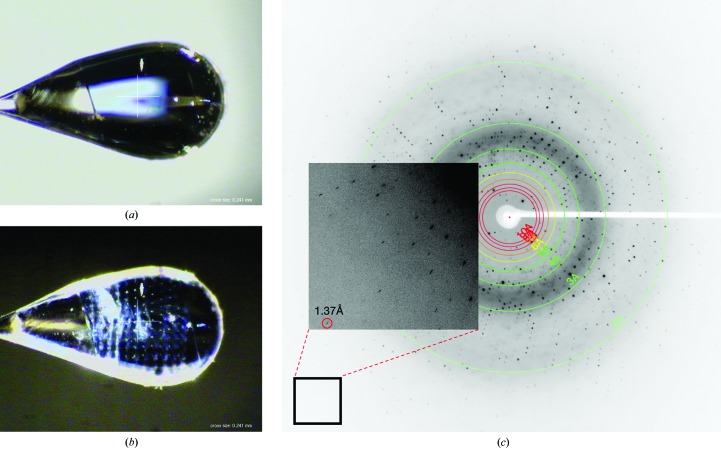
*Ax*NiR SF-ROX data collection. The crystal is shown (*a*) before and (*b*) after XFEL beam exposure. The crystal size is approximately 1 × 0.7 × 0.08 mm. Holes drilled by the X-ray laser are clearly visible corresponding to the top left to bottom right data-collection strategy. Each hole is 50 µm apart, with a 0.1° rotation between each snapshot. The XFEL diffraction setup was the same as that described by Hirata *et al.* (2014 ▸). The blue colour of the crystal shows the oxidation state of the copper site. (*c*) A diffraction image from an *Ax*NiR crystal obtained on BL3 at SACLA. The maximum visible resolution spots appear at 1.37 Å.

Large crystals of *Ax*NiR were grown for SF-ROX experiments using the as-isolated enzyme and the hanging-drop vapour-diffusion method at 4°C. 2 µl of 8 mg ml^−1^ protein solution in 10 m*M* Tris–HCl pH 7.1 was mixed with an equal volume of reservoir solution consisting of 10%(*w*/*v*) PEG 550 MME, 10 m*M* ZnSO_4_, 100 m*M* MES buffer pH 6.5. A total of 150 24-well Hampton Research crystallization plates were set up and crystals grew within three weeks. Crystals that had average dimensions of 1 × 0.8 × 0.05 mm and a two-dimensional plate morphology were selected for SF-ROX data collection (Fig. 1 ▸ *a*). One of these crystals was also used for the SRX data collection for direct comparison.

### 2.4. SRX resting state (SRX^RS^) data collection and processing   

A data set was collected from a single crystal on BL41XU at SPring-8 for comparison with the SF-ROX structure (Hasegawa *et al.*, 2013 ▸). The X-ray energy was set to 10 keV to match the SACLA beam energy. The crystal was soaked in 35%(*w*/*v*) PEG 550 MME in crystallization buffer for 60 s before cooling in liquid nitrogen. 360 images were collected using a PILATUS 6M detector, which took a total of 72 s for the full data set. The beam size was set to 44.0 µm (height) × 12.0 µm (width) with a photon flux of 1.5 × 10^13^ photons s^−1^ and an attenuation of 460 µm of Al. The data were processed using *XDS* (Kabsch, 2010 ▸) and *AIMLESS* (Evans & Murshudov, 2013 ▸) in space group *R*3. The resolution limit was cut off at 1.6 Å based on CC_1/2_ = 0.5, and 5% of the reflections were selected for use in calculating *R*_free_. The phases were determined using molecular replacement with *MOLREP* (Vagin & Teplyakov, 2010 ▸) using the *Ax*NiR monomer as the model (PDB entry 1oe1; Ellis *et al.*, 2003 ▸). Refinement was performed by *REFMAC*5 (Murshudov *et al.*, 2011 ▸) in the *CCP*4 suite (Winn *et al.*, 2011 ▸) with riding H atoms and isotropic *B* factors. The *Coot* (Emsley & Cowtan, 2004 ▸) graphics interface was used for manual model rebuilding and water addition in between each cycle of refinement. The quality of the final model was checked using *MolProbity* (Chen *et al.*, 2010 ▸). All data-processing and refinement statistics for SRX structures are given in Table 1 ▸.

### 2.5. SF-ROX data collection and processing   

SF-ROX data collection was carried out on BL3 EH4 at the SACLA XFEL (Tono *et al.*, 2013 ▸). All crystals were treated to the same cryoprotection as used during SRX^RS^ data collection and were maintained at 100 K. Single-shot diffraction images were collected using the SF-ROX data-collection strategy described by Hirata *et al.* (2014 ▸). Images were obtained from a total of 64 crystals over the course of 10 h. The X-ray energy was set to 10 keV and the pulses were of sub-10 fs duration. The powerful X-ray pulses produced by SACLA at the beam focus caused significant damage to the protein crystals. After a single exposure at the beam-focus position [beam size of 2.0 µm (height) × 1.3 (width)], some crystals cracked or were destroyed completely. Therefore, the crystals were positioned 15 mm downstream of the beam focal point, where a flux of 3 × 10^10^ photons and a beam size of 3.0 µm (height) × 1.9 µm (width) were provided. The reduced X-ray density prevented physical damage to the crystal, with only the X-ray pulse holes visible as ‘drilled holes’ (Fig. 1 ▸ *b*). The crystals were rotated 0.1° and translated 50 µm between each snapshot. The rotation step was set to approximately one-third of the crystal mosaicity determined from the SRX^RS^ data set. The camera length was set to 120 mm. 4403 single-shot images were collected in total on a Rayonix MX225-HS CCD detector with 2 × 2 binning mode corresponding to a pixel size of 78.2 µm. 3656 images with sufficient diffraction spots corresponding to good hits by the XFEL pulse were selected for downstream analyses using *DISTL* (Fig. 1 ▸ *c*; Zhang *et al.*, 2006 ▸).

The diffraction images were processed using the *CrystFEL* suite (White *et al.*, 2012 ▸). The inbuilt spot-finding algorithm *zaef* was used and indexing was carried out using *MOSFLM* (Battye *et al.*, 2011 ▸) in space group *R*3 with a 95.8% indexing rate. The inner, middle and outer integration radii were set to five, seven and eight pixels, respectively. *Ambigator* (White *et al.*, 2016 ▸) was used to resolve the indexing ambiguity, and the Bragg intensities were merged using the Monte Carlo method with frame scaling. The resolution limit was set to 1.6 Å based on a CC_1/2_ of ∼0.5 in the outer shell. The structure was built and refined as for the SRX^RS^ structure. The quality of the final model was checked using *MolProbity* (Chen *et al.*, 2010 ▸). Data-processing and refinement statistics are shown in Table 1 ▸.

## 3. Results   

### 3.1. Structure of as-isolated *Ax*NiR using BL41XU at SPring-8 (SRX^RS^)   

**Figure 2:**
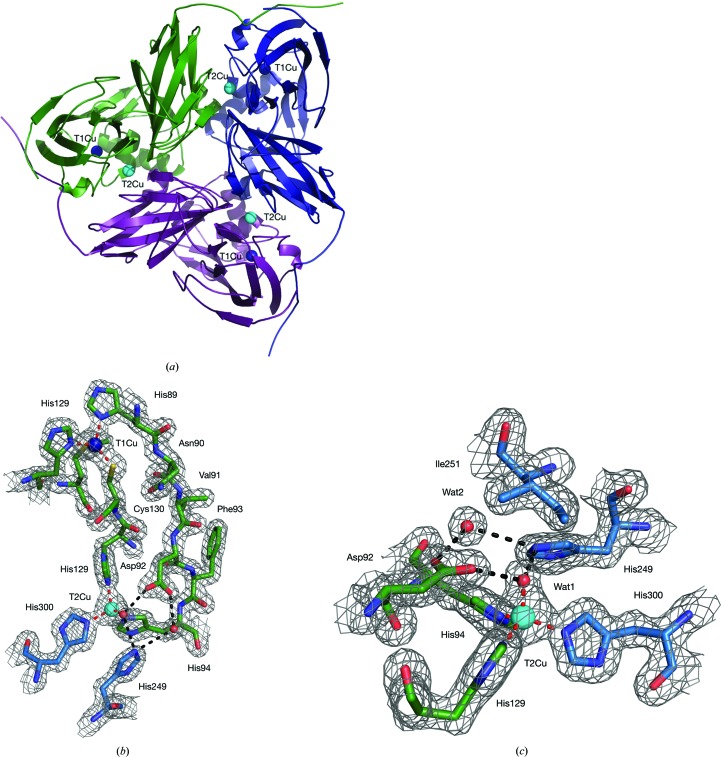
Structural organization of *Ax*NiR. (*a*) The *Ax*NiR trimer is viewed from the top down. The T1Cu is shown on the surface of the molecule, where it accepts electrons from carrier proteins. The T2Cu ion can be seen at the monomer–monomer interface. (*b*) Details of the SRX T1Cu and T2Cu sites. Electrons donated to the T1Cu are transferred along a Cys130–His129 bridge to the T2Cu. The catalytic Asp92 and His249 residues are positioned next to the substrate as the proton supply. (*c*) Resting state of the *Ax*NiR T2Cu site (SRX). The T2Cu is ligated by three histidine residues and a water ligand Wat1, which is hydrogen-bonded to two catalytic resides Asp92_CAT_ and His249_CAT_, which in turn are linked to water Wat2. Wat1 is always observed in all resting-state CuNiR structures obtained at synchrotron facilities. 2*F*_o_ − *F*_c_ electron density is contoured at the 2σ level and shown as a grey mesh. Atoms are coloured by element, with a different colour scheme used for the different chains. T1Cu is shown as a dark blue sphere, T2Cu as a cyan sphere and water molecules as small red spheres. Metal-coordinating bonds are shown as red dotted lines. Selected hydrogen bonds are shown as black dotted lines.

An SRX resting-state (SRX^RS^) data set was collected and processed to 1.6 Å resolution (Table 1 ▸). The 1.6 Å resolution SRX resting-state (SRX^RS^) global structure remains generally unchanged from the atomic resolution structure of *A. xylos­oxidans* (PDB entry 1oe1; Ellis *et al.*, 2003 ▸). The *Ax*NiR protein is trimeric, with each monomer consisting of 335 residues. A single monomer was present in the asymmetric unit and the trimer was formed from the crystal symmetry (Fig. 2 ▸ *a*). The N-terminal residue modelled as pyroglutamic acid (PCA) in PDB entry 1oe1 has no electron density in this structure and was not built (Ellis *et al.*, 2003 ▸). Electron density was not visible for the side chain of the C-terminal residue Arg336, indicating flexibility of this residue. A polyethylene glycol (PEG) molecule is bound to the His313 residue, as has previously been observed in crystal structures of *Ax*NiR crystallized in PEG 550 MME. One Zn atom is found at the boundary between two monomers, ligated by Glu195 in one monomer and by Asp167 and His165 in the neighbouring monomer, with a water ligand as the sixth ligand. Two MES molecules from the crystallization buffer are also present, hydrogen-bonded to the protein surface. 378 water molecules are present in the structure. Both the type 1 and type 2 copper sites were determined to be fully occupied based on their relative *B* factors (Fig. 2 ▸ *b*). For comparison with the atomic resolution SRX structure (PDB entry 1oe1), the structures were aligned based on the secondary structure of the NiR trimer. Both the T1Cu and T2Cu sites showed no significant changes in ligand geometry (Table 2 ▸). The fourth ligand site of the SRX^RS^ T2Cu is occupied by a water hydrogen-bonded to His249_CAT_ and Asp92_CAT_, which are in turn hydrogen-bonded to a linking water (Wat1), as observed in other cryo SRX CuNiR structures (Fig. 2 ▸ *c*; Godden *et al.*, 1991 ▸; Ellis *et al.*, 2001 ▸). The Asp92_CAT_ residue is present in the proximal configuration, as expected in the ‘substrate/product-free’ state, while the Ile251 residue is positioned to provide a steric constraint on the orientation of bound ligands (Boulanger *et al.*, 2000 ▸). The catalytic His249_CAT_ residue is further hydrogen-bonded to the carbonyl O atom of Glu273. Rotation of this residue is associated with ligand binding and also radiation-induced structural changes (Fukuda *et al.*, 2016 ▸).

### 3.2. Structure of as-isolated *Ax*NiR using serial femtosecond rotation crystallography (SF-ROX)   

**Figure 3:**
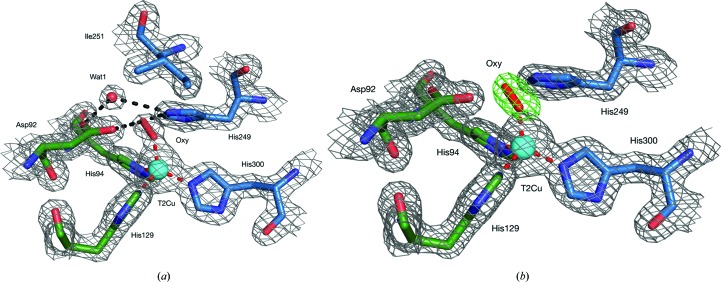
The T2Cu site as determined using femtosecond X-ray pulses. (*a*) 2*F*_o_ − *F*_c_ electron-density map of the SF-ROX *Ax*NiR T2Cu site. Electron density is contoured at 2σ around the residues and ligands of the T2Cu site as a grey mesh. An oxygen diatom is ligated to the T2Cu in place of the normally observed water molecule. The O_2_/superoxide is ligated by the catalytic residues Asp92_CAT_ and His249_CAT_, which in turn are hydrogen-bonded to the linking water. (*b*) A simulated-annealing OMIT map showing the dioxygen ligand bound to the T2Cu site. A 2*F*_o_ − *F*_c_ electron-density map contoured at 2σ in grey mesh around the residues and Cu atom is shown superimposed with a ligand *F*_o_ − *F*_c_ electron-density map contoured at 5σ in green mesh around the refined dioxygen ligand. Atoms are coloured by element, with a different colour scheme used for the different chains. T2Cu, cyan sphere; water molecules, small red spheres; oxygen diatom, red sticks. Metal-coordinating bonds are shown as red dotted lines. Selected hydrogen bonds are shown as black dotted lines.

**Figure 4:**
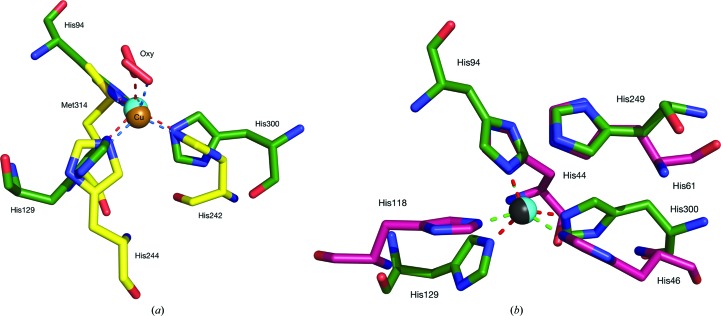
Copper-site comparison of dioxygen-binding enzymes. (*a*) Superposition of the SF-ROX *Ax*NiR resting-state T2Cu site and dioxygen-bound PHM. *Ax*NiR is shown in green and PHM is shown in yellow, with the Cu atoms shown in cyan and brown, respectively. *Ax*NiR coordinate bonds are shown in red, with PHM coordinate bonds shown in blue. PHM is from PDB entry 1sdw (Prigge *et al.*, 2004 ▸). The PHM copper has His_2_–Met coordination. Both structures have an end-on-bound dioxygen (shown in red). (*b*) Superposition of the SF-ROX *Ax*NiR resting-state T2Cu site and the reduced bovine SOD Cu site. *Ax*NiR is shown in green and Cu,Zn-SOD is shown in purple, with their Cu atoms shown in cyan and dark grey, respectively. *Ax*NiR coordinate bonds are shown in red, with SOD coordinate bonds shown in green. Bovine Cu,Zn-SOD is from PDB entry 1q0e (Hough & Hasnain, 2003 ▸). The copper sites display similar His_3_ ligation. The NiR His_CAT_ occupies a similar position to the SOD bridging imidazole.

For the SF-ROX model, the SRX^RS^ model was used for molecular replacement. The structure was determined at the same resolution as the SRX^RS^ structure: 1.6 Å (Table 1 ▸). Again, no electron density was visible for the N-terminal residue, but the C-terminal arginine did have density for the backbone and the majority of the side chain, apart from the guanidine group. Both the zinc and the MES molecules that were visible in the SRX^RS^ structure were also observed, along with 386 waters, and again both copper sites were fully occupied. Water was originally assigned as the fourth T2Cu coordinate, as is normally seen in CuNiRs. However, there was a large patch of positive density on top of the T2Cu in the SF-ROX model adjacent to the water molecule and the two catalytic residues. A second water was added manually, but the bond distance was much too short for a hydrogen bond at 1.28 Å (typically 2.4–3.2 Å). Modelling chloride, as seen in the SFX structure of *Af*NiR, originating from purification procedures and/or from the crystallization buffer also failed owing to the much stronger scattering that originates from chloride compared with the experimental electron density in our case and was incompatible with the chlorine coordination sphere. The density is also not consistent with it being assigned to NO_2_^−^ bound to the T2Cu. Furthermore, no NO_2_^−^ had been added to the crystal before cooling. The density was clearly due to a diatomic molecule that is shifted towards the linking water position from the normal binding position of NO. In view of the known superoxide dismutase activity of *Ax*NiR, O_2_ was modelled with full occupancy that satisfied the electron-density map well (Fig. 3 ▸ *a*). This is clearly demonstrated by the simulated-annealing OMIT map (Fig. 3 ▸ *b*). The O—O species bond distance was refined to 1.24 Å with *REFMAC*5 (Murshudov *et al.*, 2011 ▸) restraints, which was compatible with dioxygen superoxo/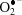 species and is very similar to the mononuclear copper active-oxygen complexes of peptidyl­glycine α-hydroxylating monooxygenase (PHM; Fig. 4 ▸ *a*; Prigge *et al.*, 2004 ▸). Structural similarity of the T2Cu site to the superoxide-binding Cu,Zn-SOD Cu site is also observed (Fig. 4 ▸ *b*). The O–O distance of 2.55 Å between the O_2_ ligand and the carboxy O atom of Asp92 probably means that the carboxy group is protonated, as O_2_ cannot provide a proton (Table 2 ▸). In CuNiR derived from *Achromobacter cycloclastes* (*Ac*NiR), His_CAT_ is protonated and hydrogen-bonded to Asp_CAT_ *via* a water molecule as revealed by atomic resolution data (Antonyuk *et al.*, 2005 ▸).

### 3.3. Comparison of SRX^RS^ and SF-ROX structures   

**Figure 5:**
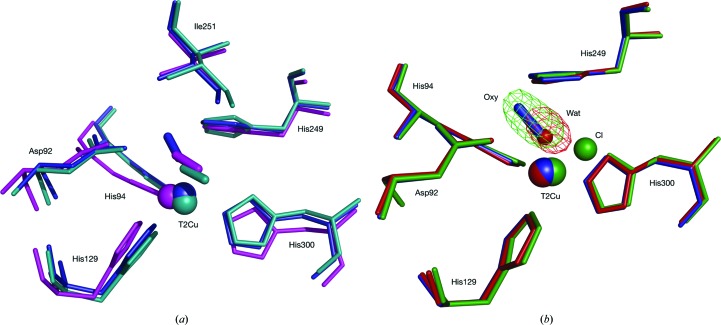
Ligand binding of T2Cu sites. (*a*) A comparison of the T2Cu ligand binding of NO by SRX (Horrell *et al.*, 2016 ▸), of O_2_ by SRX (Fukuda *et al.*, 2015 ▸) and of O_2_ by SF-ROX. The *Ax*NiR SF-ROX O_2_/superoxide is represented in blue, the *Ac*NiR SRX NO in teal and the *Gt*NiR SRX O_2_ in magenta (*Ax*NiR numbering). The teal NO and magenta O_2_ ligands in the SRX models both occupy a similar binding mode apical to the T2Cu, while the blue O_2_ ligating the SF-ROX structure is shifted significantly towards the two catalytic residues in an end-on mode. (*b*) A comparison of SRX *Ax*NiR, SF-ROX *Ax*NiR and SFX *Af*NiR resting-state T2Cu sites. The *Ax*NiR SRX structure is shown in red, the *Ax*NiR SF-ROX structure in blue and the *Af*NiR SFX structure in green (Fukuda *et al.*, 2016 ▸). The simulated-annealing *F*_o_ − *F*_c_ OMIT electron-density maps are superimposed on the oxygen and water ligands contoured at 6σ in green and red meshes, respectively. The SFX *Af*NiR resting-state T2Cu is bound by chloride ion from purification that is bound asymmetrically with respect to the His_3_ plane.

A small change of 8° was observed in the χ^2^ dihedral angle of the His249_CAT_ residue between the SF-ROX and the SRX^RS^ structures. In both structures the His294_CAT_ N^δ1^ atom only hydrogen-bonds to the Glu273 residue and the T2Cu apical ligand. This is smaller than the 20° change observed in *Af*NiR and no new hydrogen bonds are formed (Fukuda *et al.*, 2016 ▸). The appearance of the oxygen ligand is more significant owing to the novel binding mode exhibited in comparison to previously described structures with bound diatomic molecules (Fig. 5 ▸ *a*; Antonyuk *et al.*, 2005 ▸; Fukuda *et al.*, 2015 ▸). Here, we observe a mononuclear copper(II)-superoxo complex with asymmetric O–O binding. This differs from the O_2_ binding observed in *Gt*NiR, where the binding is relatively symmetric, and is more similar to NO binding after NO_2_^−^ reduction in *Ac*NiR, with O_distal_ and O_proximal_ bond distances of 2.40 and 2.13 Å, respectively, as opposed to 2.65 and 1.81 Å in *Ax*NiR (Antonyuk *et al.*, 2005 ▸; Fukuda *et al.*, 2015 ▸). This shift may be owing to the replacement of isoleucine with valine in *Gt*NiR, a thermophilic protein, that creates great steric hindrance to the ligand. The asymmetric binding nature of dioxygen observed here in *Ax*NiR shows a distinct similarity to the dioxygen binding observed in PHM (Prigge *et al.*, 2004 ▸). The end-on binding mode was compatible with the formation of a copper(II)-superoxo species, with an O—O bond distance refined to 1.23 Å based on a crystal resolution of 1.85 Å and a Cu—O—O angle of 110°. This compares favourably with the dioxygen geometry observed using SF-ROX, with an O—O bond distance of 1.24 Å and a Cu—O—O angle of 119°.

A direct comparison of the resting state of *Ax*NiR as obtained by SF-ROX and SFX (both represent ‘time-frozen’ structures, *i.e.* unaltered by the interaction with X-rays) can be made. In the SFX structure of *Af*NiR, a chloride ion is found ligated to the T2Cu (Fig. 5 ▸ *b*; Fukuda *et al.*, 2016 ▸). Apart from the changes in the T2Cu ligand, the *Af*NiR and *Ax*NiR internal water structures are very similar. A comparison of the as-isolated SF-ROX structure of *Ax*NiR and the NO_2_^−^-soaked *Af*NiR SFX structure (PDB entry 5d4i; Fukuda *et al.*, 2016 ▸) shows very little difference in structure in the active site apart from the change in ligand. Both T2Cu atoms deviate similarly from the His_3_ plane, at 0.75 and 0.77 Å, respectively. The bound NO_2_^−^ occupies a top-down, symmetrical coordination with O—Cu bond lengths of 1.93 and 2.14 Å. Again, this is linked to the position of NO viewed in *Ac*NiR *via* multiple structures collected serially from one crystal (MSOX) and is radically different from the coordination of O_2_^−^ observed here. The His_CAT_ in the NO_2_-soaked *Af*NiR SFX structure is translated 0.7 Å relative to the SF-ROX structure of *Ax*NiR and forms a second hydrogen bond to the carbonyl side chain of Thr274 (*Ax*NiR numbering) in addition to the one that they both share with Glu273. The linking water occupies the same position in both structures.

## 4. Discussion   

**Figure 6:**
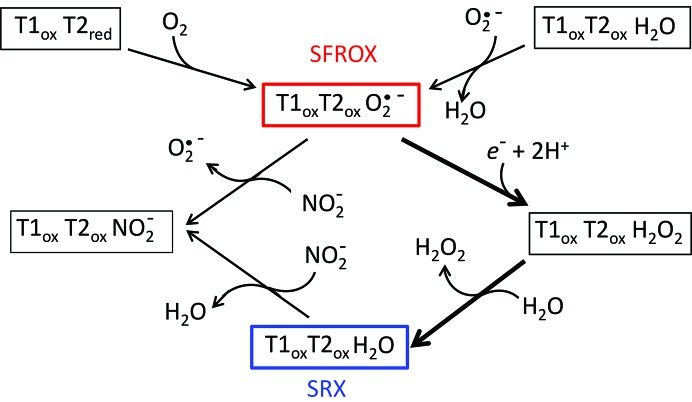
A simplified reaction scheme for the binding of dioxygen and nitrite to *Ax*NiR based on structural characterization. The superoxide species characterized by SF-ROX is stable unless a second electron is supplied. It could be arrived from two possible routes.

We have determined the structure of *Ax*NiR in a fully oxidized as-isolated state from blue crystals of *Ax*NiR by SF-ROX and compared it with the structure obtained from the same batch of crystals using conventional cryo SRX methods. The global architecture of the structures is near-identical, but the solvent-derived ligand of the T2Cu was different, with a dioxygen species in the SF-ROX structure in place of the H_2_O normally observed in SRX structures. The binding of O_2_ to a number of enzymes with mononuclear copper centres has been shown to result in oxidation of the centre to yield a copper(II)-superoxo adduct (Itoh, 2006 ▸). Since in our experiments the short duration (less than 10 fs) of the X-ray laser pulse precludes X-ray-induced chemistry, the SF-ROX structure obtained from the blue crystals is that of the copper(II) oxidation state. In the case of purified CuNiRs, the copper centres of the as-isolated enzyme are both oxidized and would not be expected to react with dioxygen. However, at an early stage of purification more reduced enzyme species are present that allow the structural characterization of stable forms of *Ac*NiR with NO_2_^−^, or NO_2_^−^ and NO, bound to the active-site T2Cu (Antonyuk *et al.*, 2005 ▸). The stability of these species was attributed to the T2Cu ion remaining reduced owing to the increase in the reduction potential of the T2Cu site to ∼1100 mV when nitrite is bound, preventing the back reaction of ET to the T1Cu centre (Ghosh *et al.*, 2009 ▸). Since neither the purified enzyme nor the crystals used for structure determination had been exposed to NO_2_^−^, these reduced species must originate from exposure to substrate/product during denitrification. In the present case, our finding of a dioxo species bound to the T2Cu ion suggests that dioxygen reacted with a reduced NiR species at an early stage in isolation of the enzyme and represents a trapped intermediate of the oxidase reaction awaiting the delivery of a second electron to enable turnover to produce H_2_O_2_. Alternatively, a superoxide radical can bind to the oxidized enzyme (Fig. 6 ▸). The stability of these ligand-bound species may arise from proton delivery to the T2Cu being compromised, preventing their further reduction.

Consistent with this proposal, microspectroscopy of *Gt*NiR during SRX data collection revealed an absorption band at 352 nm attributed to a copper(II)-superoxo species (Fukuda *et al.*, 2015 ▸). A D98N mutation of the Asp_CAT_ of *Af*NiR, in addition to decreasing the NiR activity to 1% of that of the wild-type enzyme, also shows a strongly diminished rate of O_2_ reduction, with a suggested role for the Asp_CAT_ residue in transiently stabilizing a singly protonated, two-electron-reduced copper(II)-hydroperoxo intermediate (MacPherson *et al.*, 2010 ▸). The modelled O_2_ species perfectly fits the electron density and its bond distance was refined to 1.24 Å, which is compatible with O_2_/superoxide (1.2–1.3 Å) but not peroxide (1.4–1.5 Å) (Cramer & Tolman, 2007 ▸). The O_2_ also has full occupancy, which is unusual for ligands bound to the T2Cu site of CuNiR (Fukuda *et al.*, 2015 ▸; Antonyuk *et al.*, 2005 ▸). NO binding in CuNiR is axial to the T2Cu and the same is true of O_2_ as observed in the thermophilic *Gt*NiR (Fukuda *et al.*, 2015 ▸). In the *Ax*NiR SF-ROX structure, the geometry of its binding mode is unique in that it is not bound directly above the T2Cu but is translated towards the two catalytic residues. The O—O bond distance of 1.24 Å is very similar to the O—O distance of 1.23 Å observed in the 1.85 Å resolution structure of PHM, where it was assigned to a copper(II)-superoxo complex. This structure is not observed in the SRX structures of CuNiRs since X-ray photoreduction of the T1Cu results in its conversion to H_2_O_2_ and its subsequent replacement by H_2_O, which is observed in the SRX^RS^ T2Cu site. The X-ray-induced O_2_ species observed in the thermophilic *Gt*NiR SRX structure that has a different position/orientation may represent an intermediate in the conversion to H_2_O_2_. The ubiquitous presence of H_2_O in SRX structures of these CuNiRs with oxidase activity can be explained if radiolysis-induced turnover results in reduction of the bound dioxygen species to H_2_O_2_, which is then replaced by water (Fig. 6 ▸).

Our results warrant a re-examination of many of the spectroscopic studies performed on aerobically purified and maintained enzymes. The SF-ROX structure with an O_2_ species provides a clear rationale for the SOD activity of this enzyme, which needs to be systematically explored in this enzyme family. The focus on the nitrite-reduction function of these enzymes has so far limited the characterization of these proteins as oxidases, although the oxidase activity of several nitrite reductases has been noted. Of all the NiRs for which structures are known, the NiR from *Nitrosomonas europaea* (*Ne*NiR), an organism that denitrifies under aerobic conditions, has been shown to have no oxidase activity. An SRX structure of this enzyme showed that the substrate-access channel to the active site is more restricted compared with those observed in typical CuNiRs (Lawton *et al.*, 2013 ▸). The ability of femtosecond X-ray crystallography to provide structural diffraction data from single-shot pulses on a more rapid timescale than the molecular dynamics has allowed the determination of a true resting state for this important enzyme and its comparison with the ‘time-frozen’ structure of the substrate (nitrite)-bound species. This unique capability of XFEL radiation is likely to have a major impact on the structure-based mechanism of complex biological systems, as has been demonstrated for O=O bond formation in PSII (Suga *et al.*, 2017 ▸).

## Supplementary Material

PDB reference: CuNiR, SF-ROX structure, 5onx

PDB reference: SFX structure, 5ony

Supplementary figures: AxNiR SF-ROX data processing and pairwise alignment of AfNiR and AxNiR.. DOI: 10.1107/S2052252517016128/ec5005sup1.pdf

## Figures and Tables

**Table 1:** Data-processing and refinement statistics Values in parentheses are for the highest resolution shell.

	SF-ROX	SRX^RS^
Data collection		
Space group	*R*3:*H*	*R*3:*H*
Unit-cell parameters		
*a*, *b*, *c* (Å)	89.97, 89.97, 143.56	90.35, 90.35, 143.58
α, β, γ (°)	90, 90, 120	90, 90, 120
Resolution (Å)	19.84–1.60 (1.63–1.60)	47.86–1.60 (1.63–1.60)
*R*_split_ † (%)	18.6 (77.2)	
*R*_p.i.m._ (%)		7.5 (29.6)
〈*I*/σ(*I*)〉	4.7 (2.0)	5.1 (1.2)
CC_1/2_ ‡	0.975 (0.502)	0.981 (0.782)
Completeness (%)	100.0 (100.0)	98.0 (97.8)
SFX multiplicity§	105.5 (47.0)	
Redundancy		3.3 (3.1)
Wilson *B* factor (Å^2^)	26.1	23.6
Refinement		
No. of reflections	57138 (5685)	56521 (2791)
*R*_work_/*R*_free_ (%)	18.5/22.6	19.0/22.3
No. of atoms		
Protein	2594	2578
Ligand/ion	42	40
Water	386	378
*B* factors (Å^2^)		
Protein	29.0	27.4
Cu	26.1	25.9
Zn	31.0	28.4
MES	49.2	46.0
PEG	39.1	44.5
Dioxygen	32.1	
Water	40.3	39.0
R.m.s. deviations		
Bond lengths (Å)	0.015	0.018
Bond angles (°)	1.758	1.900
PDB code	5onx	5ony

† *R*_split_ = 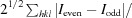 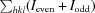.

‡ CC_1/2_ is the correlation coefficient between two half data sets (Karplus & Diederichs, 2012 ▸).

§ SFX multiplicity refers to several partial intensity measurements and not to fully integrated intensities, which is referred to as redundancy.

**Table 2:** Copper-site geometries

	SF-ROX	SRX^RS^	SRX^RS^ (PDB entry 1oe1)
T1Cu site distances (Å)			
T1Cu–His89 N^δ1^	1.96	2.11	2.02
T1Cu–Cys130 S^γ^	2.08	2.11	2.20
T1Cu–His139 N^δ1^	1.96	2.02	2.03
T1Cu–Met144 S^δ^	2.72	2.75	2.45/4.26
T1Cu site angles (°)			
His89 N^δ1^–Cu–Cys130 S^γ^	131.7	125.6	121.5
His89 N^δ1^–Cu–His139 N^δ1^	99.9	103.6	100.5
His89 N^δ1^–Cu–Met144 S^δ^	84.3	82.4	88.4/87.5
Cys130 S^γ^–Cu–His139 N^δ1^	119.5	122.5	113.9
Cys130 S^γ^–Cu–Met144 S^δ^	105.8	106.2	113.5/107.1
His139 N^δ1^–Cu–Met144 S^δ^	109.0	108.4	116.4/124.7
T2Cu site distances (Å)			
T2Cu–His94 N^δ2^	2.04	2.04	1.96
T2Cu–His129 N^δ2^	2.02	2.01	2.00
T2Cu–His300 N^δ2^	2.06	2.09	2.00
T2Cu–Wat1		1.72	1.98
T2Cu–Oxy^1^	2.65		
T2Cu–Oxy^2^	1.81		
Asp92 O^δ2^–Oxy^1^	2.55		
His249 N^δ2^–Oxy^1^	2.24		
T1Cu site angles (°)			
His94 N^δ2^–T2Cu–His129 N^δ2^	104.8	108.2	110.9
His94 N^δ2^–T2Cu–His300 N^δ2^	100.0	106.4	103.6
His129 N^δ2^–T2Cu—His300 N^δ2^	112.5	108.6	108.9
T2Cu–His_3_ ligand plane (Å)	0.75	0.74	0.72
