# Supplementary material for: An unprecedented dioxygen species revealed by serial femtosecond rotation crystallography in copper nitrite reductase
Source: IUCrJ. 2018 Jan 1;5(Pt 1):22–31. doi: 10.1107/S2052252517016128 (PMC5755574; doi:10.1107/S2052252517016128)
Supplement: Supplementary file 1 [file m-05-00022-sup1.pdf]

IUCrJ

Volume 5 (2018)

Supporting information for article:

An unprecedented dioxygen species revealed by serial femtosecond rotational crystallography in copper nitrite reductase

Thomas P. Halsted, Keitaro Yamashita, Kunio Hirata, Hideo Ago, Go Ueno, Takehiko Tosha, Robert R. Eady, Svetlana V. Antonyuk, Masaki Yamamoto and S. Samar Hasnain

Supplementary information

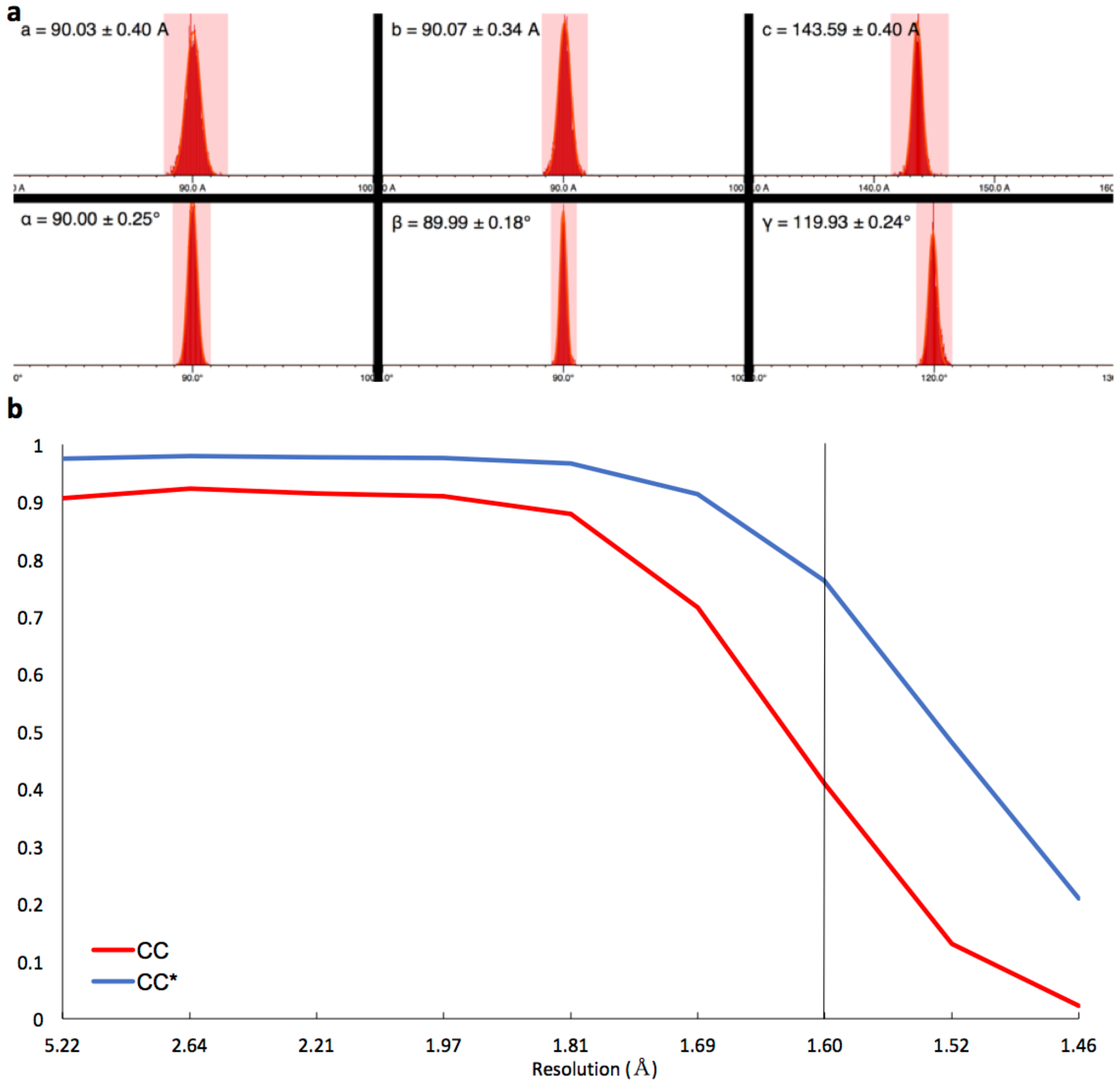

Figure S1 AxNiR SF-ROX data processing**. a.** The distribution of unit cell parameters displayed by *cell explorer*. The unit cell lengths and angles from individually indexed snapshots from *indexamajig* displayed as a distribution (White *et al.*, 2016). Red colouring represents these parameters that fall into a H centred space group. **b.** The correlation coefficient statistics for AxNiR, SF-ROX data processing. The CC and CC* vs the resolution during data processing. Data was cut-off at 1.6 Å based on $CC_{1/2} = 0.5$. CC* is calculated from $CC_{1/2}$. $CC^* = [2\,CC_{1/2}/(1 + CC_{1/2})]^{1/2}$ (Karplus & Diederichs, 2012).

```
AxNiR           1 -----DADKLPHTKVTLVAPPQVHPHEQATKSGPKVVEFTMTIEEKKMVI     45
                       :...||..||.||.||.||.|.|..:.|||||||||.|||||:||
AfNiR           1 MATAAEIAALPRQKVELVDPPFVHAHSQVAEGGPKVVEFTMVIEEKKIVI     50

AxNiR          46 DDKGTTLQAMTFNGSMPGPTLVVHEGDYVQLTLVNPATNAMPHNVDFHGA     95
                  ||.||.:.||.|||::|||.:|||:.||::|||:||.||.:.||:|||.|
AfNiR          51 DDAGTEVHAMAFNGTVPGPLMVVHQDDYLELTLINPETNTLMHNIDFHAA    100

AxNiR          96 TGALGGAKLTNVNPGEQATLRFKADRSGTFVYHCAPEGMVPWHVVSGMSG    145
                  ||||||..||.:||||:..|||||.:.|.|||||||.|||||||||||:|
AfNiR         101 TGALGGGGLTEINPGEKTILRFKATKPGVFVYHCAPPGMVPWHVVSGMNG    150

AxNiR         146 TLMVLPRDGLKDPQGKPLHYDRAYTIGEFDLYIPKGPDGKYKDYATLAES    195
                  .:|||||:||.|.:||.|.||:.|.:||.|.|:|:..:||||.|....::
AfNiR         151 AIMVLPREGLHDGKGKALTYDKIYYVGEQDFYVPRDENGKYKKYEAPGDA    200

AxNiR         196 YGDTVQVMRTLTPSHIVFNGKVGALTGANALTAKVGETVLLIHSQANRDT    245
                  |.|||:|||||||:|:||||.||||||..|:||.|||.||::||||||||
AfNiR         201 YEDTVKVMRTLTPTHVVFNGAVGALTGDKAMTAAVGEKVLIVHSQANRDT    250

AxNiR         246 RPHLIGGHGDWVWETGKFANPPQRDLETWFIRGGSAGAALYTFKQPGVYA    295
                  ||||||||||:||.||||..||..|.|||||.||:||||.|||:|||:||
AfNiR         251 RPHLIGGHGDYVWATGKFNTPPDVDQETWFIPGGAAGAAFYTFQQPGIYA    300

AxNiR         296 YLNHNLIEAFELGAAGHIKVEGKWNDDLMKQIKAPAP--IPR    335
                  |:|||||||||||||.|.||.|:||||||..:.||:.  :||
AfNiR         301 YVNHNLIEAFELGAAAHFKVTGEWNDDLMTSVLAPSGTLVPR    342
```

Figure S2 AfNiR and AxNiR pairwise alignment. Compared using the EBLOSUM62 matrix. Similarity score of 77.5%.

References

Karplus, P. A. & Diederichs, K. (2012). *Science (80-.)*. **336**, 1030–1033.

White, T. A., Mariani, V., Brehm, W., Yefanov, O., Barty, A., Beyerlein, K. R., Chervinskii, F., Galli, L., Gati, C., Nakane, T., Tolstikova, A., Yamashita, K., Yoon, C. H., Diederichs, K. & Chapman, H. N. (2016). *J. Appl. Crystallogr.* **49**, 680–689.
